# BARRIERS AND SOLUTIONS TO IMPROVE AGE-RELATED SERVICE ACCESS IN UTAH: A QUALITATIVE STUDY

**DOI:** 10.1093/geroni/igz038.3238

**Published:** 2019-11-08

**Authors:** Alex T Schiwal, Elizabeth B Fauth

**Affiliations:** 1 Center for Persons with Disabilities - Utah State University, Logan, Utah, United States; 2 Utah State University, Logan, Utah, United States

## Abstract

Utah is projected to be in the top 10 states for growth in the aging population, but it is among the most rural. Local and regional contexts guide policy and practice, and these perspectives will inform solutions as more older adults require services in rural and other under-served areas in the coming decades. Guided by Bronfenbrenner’s Process-Person-Context-Time model, this study used a qualitative participatory research orientation involving stakeholders in Utah’s aging service system in order to identify local barriers and solutions to accessing rural aging services. The stakeholders included service providers, caregivers, older adults, state-administrators, and other community members. There were 3 male and 7 female participants ranging in age from 40 to 80. Thematic analysis revealed that communities faced barriers common to rural areas (local service insufficiencies, distance and time concerns, systemic issues such as healthcare and ageism, finances - both personal and programmatic were deemed a recurrent barrier, in addition to transportation issues. However, participants reported assets in rural areas, such as a strong sense of belonging in the community and creative problem solving. Solutions for improving access to age-related services included strategies for making information more available, publicized, and centralized and increasing access to telehealth or internet-delivered services and health information. These barriers and solutions were nested across the levels of context in Bronfenbrenner’s model, with both person, time, and in interactions (processes) having influence, but localized analysis of the barriers is necessary to ensure that the solutions are appropriate in a specific context.

